# Role of Montelukast in Asthma and Allergic rhinitis patients

**DOI:** 10.12669/pjms.36.7.2657

**Published:** 2020

**Authors:** Faisal Faiyaz Zuberi, M. Amir Haroon, Abdul Haseeb, Shafi Muhammad Khuhawar

**Affiliations:** 1 Dr. Faisal Faiyaz Zuberi, M.B.B.S., F.C.P.S (Med)., F.C.P.S (Pulm), FCCP. Associate Professor Pulmonology, Head of Chest Unit-II, Dow University of Health Sciences, Karachi, Pakistan; 2 Dr. M. Amir Haroon, M.B.B.S., D.T.C.D., M.D. Pulmonologist and Consultant Physician, Sindh Government Hospital Liaquatabad, Karachi, Pakistan; 3 Dr. Abdul Haseeb, M.B.B.S., D.T.C.D (UK)., M.C.P.S (Chest). Consultant Chest Specialist, Jamal Noor Hospital, Karachi, Pakistan; 4 Dr. Shafi Muhammad Khuhawar, M.B.B.S., F.C.P.S., M.C.P.S. Associate Professor, Pulmonology, Ghulam Muhammad Mahar Medical College, Sukkur, Pakistan

**Keywords:** Allergic rhinitis, Asthma, Hypersensitivity, Montelukast, Quality of life

## Abstract

**Objectives:**

Our objective was to evaluate the effect of Montelukast on the symptoms of asthma and allergic rhinitis (AR), assess its effect on the individual quality of life (QoL), and estimate the proportion of participants having adverse effects.

**Methods:**

This prospective, open-label study conducted at Dow University of Health Sciences, Ankle Saria Hospital and Sindh Government Hospital Liaquatabad, Karachi, from August 2018 to September 2019, included patients aged >18 years with a clinical diagnosis of Asthma, AR, or both. Patients were given a 10 mg Montelukast tablet each day and then called for follow-up in the fourth week, where the questions related to the improvement in the symptoms of asthma or AR were asked. Patients were also asked about the improvement in QoL and any adverse effects.

**Results:**

A total of 694 patients were registered of which 138(19.8%) had AR, 294(42.4%) had asthma, while 273(39.3%) had both. Mean age was 41.1±14.63 years and 352 (50.7%) were male and 342(49.3%) were females. On a follow-up visit, there was a sufficient improvement in 351 asthmatics (63.9%), and 288 patients with AR (70.1%) overall, strong or marked improvement in the day (n=342,62.3%) and night time (n=331,60.3%) asthma symptoms. Overall improvements in QoL were very good or good in 419 patients. Montelukast was well-tolerated here with adverse effects (like abdominal discomfort, fever, fatigue, headache, rash, and upper respiratory tract symptoms) seen in 125 patients (18.01%).

**Conclusion:**

Montelukast was very effective in improving the symptoms and QoL of the individuals suffering from asthma and/or AR.

## INTRODUCTION

Asthma and Allergic Rhinitis (AR) are the disorders affecting a large population of our society.^1^ Both of these conditions are often observed together in the affected individuals, having mutual pathophysiology. Several mechanisms have been suggested to elaborate on their disease process with the involvement of inflammatory cells like mast cells and eosinophils, inflammatory mediators like leukotrienes, histamine, and tryptase.^2^ Asthma is a chronic inflammatory disorder of airways, presenting with variable symptoms related to the inflammation and hyper responsiveness of airways, while AR can present with rhinorrhea, enlargement of nasal turbinates and tenderness, conjunctival injection, and pallor.^3^,^4^ It is managed through minimizing the exposure to the allergen, pharmacotherapy, and immunotherapy.^5^

AR tops the list of the most commonly associated comorbidities with asthma as reported in the literature that 80% asthmatics also carry AR as a coexistent “ghost” diagnosis which in most cases remains underdiagnosed.^6^ A study from the United States reported AR symptoms in 72% of individuals with asthma, and interestingly 53% of these subjects were undiagnosed with AR. Epidemiologically, overall the existence of asthma is 30% in patients primarily diagnosed with AR, while that of AR is 70% in asthmatics. A similar kind of data from a study conducted in Japan reported the existence of AR in as many as 67.3% asthmatics.^7^-^9^

Montelukast, a Leukotriene (LT) antagonist, has a therapeutic role in the treatment of Asthma and AR by acting on Cysteinyl leukotriene-1 and 2 receptors. It is a widely used drug that was first approved in 1998 for use in the United States, indicated mostly in the prophylaxis and treatment of asthma, including the prevention of exercise-induced bronchoconstriction and AR with a recommended oral dosage of 10mg once-daily in adults.^10^

Montelukast effectively improves the QoL by addressing the symptoms in the patients, proving to be a good replacement of drugs like inhaled corticosteroids (ICS) and long-acting beta2 agonists (LABA). As per a study published in 2019, it plays a great role in the improvement of QoL by addressing the symptoms potently, as compared with a placebo group.^11^

Asthma and AR lie in the category of conditions that need lifelong therapy because of the symptomatic relapses, which is a cause as well as a reason for the lack of compliance. A patient friendly mode of management was the need of the hour since so long. Montelukast came as the answer, a single-dose therapy along with many other advantages. We have focused on analysing the role of montelukast in two of the hypersensitivity disorders, along with sorting out all the pros of using this drug and assessing the traits that make it a first-line therapy in the aforementioned indications.

## METHODS

This is a prospective, open-label study conducted at Dow University of Health Sciences, Ankle Saria Hospital and Sindh Government Hospital Liaquatabad Karachi, Pakistan, from August 2018 to September 2019. Both males and females, aged >18 years with a clinical diagnosis of Asthma or AR giving informed consent were included in the study. Pregnant or breast-feeding patients, those having a history of previous adverse reactions to montelukast, history of hyper-eosinophilic disorder other than an atopic disease, or any significant active pulmonary pathology other than asthma were excluded. The study was approved by the institutional review board (No.: DUHS/IRC/2018-003). The trial was also registered at www.clinicaltrials.gov. (Identifier: NCT03380975).

When patients signed the informed consent, a brief history was taken at registration. They were asked about the diagnosis and its symptoms, either having asthma, AR, or both. The severity of asthma was divided into categories of intermittent and persistent (mild, moderate, and persistent) according to recent guidelines.^12^ While AR was categorized as intermittent or persistent based on the duration of symptoms.^13^ Individual quality of life (QoL), assessment about sleep, work, everyday life, and physical activity was done at registration.

Patients were given a 10 mg Montelukast tablet (Aireez®), each day and then called for follow-up in the fourth week. On a follow-up visit, general improvement in asthma and AR symptoms, improvement of day and night-time asthma symptoms, and specific improvement in AR symptoms were evaluated. General improvements were categorized as very good, good, satisfactory, sufficient, or not sufficient. Specific improvements in symptoms or QoL domains were categorized as strong, marked, moderate, or none. Patients were also asked about the improvement in QoL and any adverse effects occurring during the therapy. All adverse events occurring during the study period were recorded.

Data were entered and analysed by using SPSS version 23.0, where frequency and percentages were calculated for gender, family history, the severity of asthma and AR, concomitant medications usage, symptoms, Qol categories, adverse effects of montelukast, and improvement in symptoms and QoL.

## RESULTS

A total of 694 patients were included in the study from August 2018 to September 2019 after taking informed consent. In terms of diagnosis, 138(19.8%) had AR, 294(42.4%) had asthma, while 273(39.3%) had both. The mean ± SD of age was 41.1±4.63 years which included 50.7% males and 49.3% females. The majority of participants (62.4%, n=433) had no family history of asthma. When the severity of asthma and AR were assessed, most of them had a persistent disease with further categorization as shown in Table-I. Montelukast tablet was given to all the patients despite their ongoing medications, details of which are presented in Table-I.

**Table-I:** Patient baseline characteristics at week 4 (n=694).

Characteristic	n (%)
*Gender*	
Male	352(50.7)
Female	342(49.3)
Duration (Mean ± SD) of disease	31.08 ± 61.69
Family History of Asthma	204(29.4)
*Diagnosis*	
Asthma alone	294(42.4%)
Allergic rhinitis alone	138(19.8%)
Asthma and Allergic rhinitis	273(39.3%)
*Asthma (n=549)*	
Intermittent	185(33.7)
Mild	203(36.9)
Moderate	161(29.3)
*Allergic Rhinitis (n=411)*	
Intermittent	243(59.1)
Persistent	169(41.1)
*Concomitant Medications*	
Antileukotrienes	286(41.2)
Inhaled Corticosteroids	217(31.3)
Oral Corticosteroids	140(20.2)
Long-acting inhaled beta2-agonists	81(11.7)
Short-acting inhaled beta2-agonists	42(6.1)
Short-acting oral beta2-agonists	49(7.1)
Ipratropium	163(23.5)
Theophylline	206(29.7)
Others	32(4.6)
Average inhaler Puff per day ± SD	2.60±1.03

Almost the entire study population showed both the day and night-time symptoms on presentation, with cough being the most prevalent one. On a follow-up visit, there was a sufficient improvement in 351 asthmatics (63.9%), strong or marked improvement in the day (n=342,62.3%) and nighttime (n=331,60.3%) asthma symptoms (Table-II).

**Table-II:** Symptoms of patients with asthma before and after administration of montelukast at Week 4 (n=549).

Characteristic	n (%)
*On presentation*	
*Day-time asthma symptoms*	
Cough	528(95.1)
Wheezing	440(79.3)
Chest tightness	382(68.8)
Shortness of breath	322(58.0)
Others	1(0.1)
*Night-time asthma symptoms*	
Cough	507(91.4)
Shortness of breath	407(73.3)
Nocturnal awakening	266(47.9)
Others	2(0.2)
*On follow-up*	
*Improvement in day symptoms of asthmatics*	
Strong	233(42.4)
Marked	109(19.8)
Moderate	78(14.2)
None	4(0.7)
*Improvement in night symptoms of asthmatics*	
Strong	196(35.7)
Marked	135(24.6)
Moderate	77(14.0)
None	7(1.2)
*Asthma overall improvement*	
Very good	182(33.1)
Good	169(30.7)
Satisfactory	45(8.1)
Sufficient	23(4.1)
Not sufficient	5(0.7)

The symptoms of AR were variable including sneezing, runny nose, nasal congestion, watery eyes, and red/burning eyes. On a follow-up visit, there was a sufficient improvement in 288 patients of AR (70.1%) (Table-III). Overall improvements in QoL were very good or good in 419 patients (Table-IV).

**Table-III:** Symptoms of patients with Allergic Rhinitis before and after administration of Montelukast at week 4.

Characteristic		n (%)
*On presentation*		
Allergic rhinitis symptoms	Sneezing/itching	356(64.1)
	Runny nose	310(55.9)
	Nasal congestion	269(48.5)
	Watery eyes	145(26.1)
	Red/burning eyes	119(21.4)
	Others	-
*On follow-up*		
Sneezing	Strong	94(22.9)
	Marked	72(17.5)
	Moderate	89(21.6)
	None	61(14.8)
Runny nose	Strong	102(24.8)
	Marked	62(15.1)
	Moderate	37(9.0)
	None	107(26.0)
Nasal	Strong	80(19.5)
	Marked	68(16.5)
	Moderate	62(15.1)
	None	94(22.9)
Watery eyes	Strong	79(19.2)
	Marked	51(12.4)
	Moderate	32(7.8)
	None	119(28.9)
Red eye	Strong	82(19.9)
	Marked	43(10.5)
	Moderate	29(7.1)
	None	121(29.4)
Allergic rhinitis overall improvement	Very good	82(19.9)
	Good	130(31.6)
	Satisfactory	58(14.1)
	Sufficient	18(4.4)
	Not Sufficient	6(1.4)

**Table-IV:** Individual Quality of life assessment before and after administration of Montelukast at week 4

Characteristic		n (%)
*On presentation*		
Difficulty sleeping	Mild	391(58.6)
	Moderate	238(35.7)
	Severe	38(5.7)
Difficulty with job	Mild	336(48.7)
	Moderate	330(47.8)
	Severe	24(3.7)
Difficulty with everyday life	Mild	341(49.6)
	Moderate	315(45.8)
	Severe	32(4.7)
Limitations to daily activities	Mild	268(39.4)
	Moderate	306(44.9)
	Severe	107(15.7)
*Follow-up visit*		
Sleeping	Strong	158(29.1)
	Marked	94(17.3)
	Moderate	81(14.9)
	None	210(38.7)
Job	Strong	153(28.2)
	Marked	92(16.9)
	Moderate	150(27.6)
	None	148(27.3)
Everyday	Strong	118(21.7)
	Marked	124(22.8)
	Moderate	123(22.7)
	None	178(32.8)
Daily activities	Strong	137(25.2)
	Marked	113(20.8)
	Moderate	75(13.8)
	None	218(40.1)
Overall assessment	Good	336(61.9)
	Satisfactory	187(34.4)
	Reduced	20(3.7)
Improvement	Very good	195(35.9)
	Good	224(41.3)
	Satisfactory	81(14.9)
	Sufficient	35(6.4)
	Not Sufficient	8(1.5)

As per our results, Montelukast was well-tolerated here since in this large group of patients, 125 patients (18.01%) had one or more adverse effects reported including abdominal discomfort (2.6%, n=18), fever (2.2%, n=15), fatigue (5.2%, n=36), headache (5.6%, n=39), rash (1%, n=7), and symptoms of upper respiratory tract infection (1.4%, n=10).

## DISCUSSION

The participants involved in our study showed a marked improvement in their QoL which was significantly affected before the commencement of the treatment with montelukast. Overall, a strong improvement was observed in the day, night and overall symptoms of asthma. Moreover, the majority of participants also showed improvement in AR symptoms on the follow-up i.e. sneezing, cough, nasal, and ocular ones.

Our study is similar to the work of Philip G et al., where marked improvement was observed in nasal and ocular symptoms after the two weeks treatment with montelukast 10 mg, the same study also concluded that this significant reduction in the symptoms of AR imposed a positive impact on asthma-related problems of the patient who were dealing with both the disorders.^14^ Montelukast particularly has a positive impact on cough and all the discomforts associated with it,^15^ and this is quite evident in our patients as well.

After the computation of results, headache was found to be the most prevalent among all the adverse effects of this LT antagonist. Findings of Haarman MG et al. somewhat validates this as they also reported headache as the most common adverse effect along with others like abdominal pain, aggression, abnormal behaviour, rash, and muscle spasm.^16^

The mainstay in the management of asthma has always been ICS with or without LABA. However, many asthmatics were not in the complete symptom-free state after the use of this combination. Thus the Global initiative for Asthma came up with upgrading the guidelines and included add-on therapy with LT modifiers in the management of asthma.^17^ These comprise of two main groups of CysLT1R antagonist (like montelukast, pranlukast, and zafirlukast) and 5-lipoxygenase inhibitors (like zileuton).^18^ As far as AR is concerned, montelukast maintains the balance in nitric oxide production, imbalance of which is believed to be one of the contributors in AR pathogenesis.^16^ As computed in our results, other authors have also demonstrated a good role of LT receptor antagonists in the management of asthma and AR symptoms. Once-daily oral dosage of montelukast significantly improves the airway function in asthmatics.^19^ Similarly, it also proves beneficial in reducing daytime ocular symptoms with a delayed impact on night-time symptoms of AR.^20^

There are several reasons that make montelukast stand out as a part of the treatment regimen of asthma and AR and the topmost of them is the ease in compliance for the patient as it is far easier to use a drug once-daily orally in comparison with other drugs. Secondly, this also shortens the extended side-effect profile (like that after long-term steroid usage).

### Limitations of the study

Although this study has tried to cover the role of montelukast in the treatment of asthma and AR but there are some limitations that can be listed in order to get worked on in the future, as the evaluation of short and long-term side effects associated with the use of montelukast, contraindications, toxicities, and continuous monitoring of the patient while on treatment. There was no control group or a placebo drug to compare the effects and be sure that the outcome is only due to the tested drug. Further research is needed in order to emphasize the importance and safety of this drug. Moreover, there is some gap in research regarding the role of montelukast in the treatment of several other allergic disorders to consolidate the theories behind the mechanisms and adverse effects of its use in these conditions.

## CONCLUSION

Montelukast is effective in improving the symptoms and QoL of the individuals suffering from asthma and Allergic Rhinitis.

### Authors’ Contributions:

**FFZ:** Worked on concept and design of study and questionnaire and he is also the responsible and accountable for the accuracy or integrity of the work.

**MA, AH, SM:** Contributed in data collection and reviewed the paper. They are also responsible and accountable for the accuracy and integrity of the work

All authors have read and approved the final draft of the manuscript.
